# A Comparative Study of Fault Diagnosis for Train Door System: Traditional versus Deep Learning Approaches

**DOI:** 10.3390/s19235160

**Published:** 2019-11-25

**Authors:** Seokju Ham, Seok-Youn Han, Seokgoo Kim, Hyung Jun Park, Kee-Jun Park, Joo-Ho Choi

**Affiliations:** 1 Department of Aerospace & Mechanical Engineering, Korea Aerospace University, Goyang-City 10540, Korea; tjrwn8098@naver.com (S.H.); sgkim@kau.kr (S.K.); phj921029@kau.kr (H.J.P.); 2 Urban Transit Research Group, Korea Railroad Research Institute, Uiwang-City 16105, Korea; syhan@krri.re.kr (S.-Y.H.); kjpark@krri.re.kr (K.-J.P.); 3 School of Aerospace & Mechanical Engineering, Korea Aerospace University, Goyang-City 10540, Korea

**Keywords:** train door, fault diagnosis, traditional feature extraction, convolutional neural network

## Abstract

A fault diagnosis of a train door system is carried out using the motor current signal that operates the door. A test rig is prepared, in which various fault modes are examined by applying extreme conditions, as well as the natural and artificial wears of critical components. Two approaches are undertaken toward the fault classification for comparative purposes: one is the traditional feature-based method that requires several steps for the processing features such as signal segmentation, the extraction of time-domain features, selection by Fisher’s discrimination, and K-nearest neighbor. The other is the deep learning approach by employing the convolutional neural network (CNN) to skip the hand-crafted features extraction process. In the traditional approach, good accuracy is found only after the current signal is segmented into the three velocity regimes, which enhances the discrimination capability. In the CNN, superior accuracy is obtained even by the original raw signal, which is more convenient in terms of implementation. However, in view of practical applications, the traditional approach is more useful in that the features processing can be easily applied to assess the health state of each fault and monitor the progression over time in the real operation, which is not enabled by the deep learning approach.

## 1. Introduction

The metro train system, which is a primary means of city transportation, is composed of many subsystems to keep the train operation safe and reliable, but they are often prone to failures during its operation. Among these, the train door is one of critical subsystems that can cause service delay or breakdown, leading to the increased cost of operation and maintenance [1]. According to Bombardier experience feedback, 30% to 40% of operating train failures occur in the train door systems [2]. In order to prevent these failures, recent study is being given to the fault detection and diagnosis by exploiting the sensor signals measured during its operation. In this area, there have been two approaches: model-based and data-driven. In the model-based approach, which utilizes a mathematical model describing the door dynamics, several studies have been attempted such as modeling the door mechanism by a simple ball screw table [3], modeling the motor dynamics by ordinary differential equations [4], and bond graph modeling to describe a train door mechatronic subsystems [5]. However, the train door system contains many components interconnected with various uncertainty, which makes the modeling approach of limited value in the diagnosis.

To overcome these problems, data-driven approaches have been investigated more dominantly. However, in the train door application, very little study is found in the literature. For the train plug door, the audio sensor signals are used to classify faults by using the empirical mode decomposition (EMD) and support vector machine (SVM) [6]. The health monitoring method is proposed by the resistance analysis of the motor current signals during the door movement for the two cases: internal fault by the bent screw and insufficient lubrication, and external fault by pushing of passengers and obstruction. Principal component analysis (PCA) is employed to construct the health indicators [7]. Expanding the survey to the similar systems with electric reciprocating mechanisms, more works are found in the literature. In the diagnosis of an elevator door, several types of signal ranging from motor current, encoder, four switches, and vibration are used to extract various features and classify faults by applying the wavelet packet decomposition (WPT) and logistic regression [8]. In the study of a railway point machine, the motor current signal is used for the health assessment of various failure modes by employing self-organizing maps (SOM) [9] or a series of steps including statistical feature extraction, principal component analysis (PCA), and SOM [10]. In reference [10], audio sensors are used to extract features given by the mel-frequency cepstrum coefficients (MFCCs), and SVMs are applied to diagnose faulty condition. In the other literature, the similarity of the motor current signal between the normal and fault is used for the fault diagnosis, in which the dynamic time warping [11] or similarity function and fuzzy c-means [12] are employed to detect abnormal shapes and identify fault types. As such, the data-driven approaches typically involve the process of features extraction and selection, which requires a good level of domain expertise. Regarding the features selection, more information can be found in reference [13]. 

Recently, a new approach by the deep learning techniques such as the convolutional neural network (CNN) has received great attention due to the direct use of the raw signal with minimal engagement of domain-specific skills. Among many others, the CNN has shown good performance in the video action recognition [14]. The CNN has also extensive applications in the machine health monitoring, as addressed in [15]. However, applications to the train door or similar machines are rare in the literature. Fault diagnosis of high-speed railway point machines is carried out using the converted 2D images of current signals in the CNN [16]. 

As seen from the above, the main research focus of the traditional data-driven approach has been to find out good features for the fault classification by using the external add-on sensors such as sound or vibration or internal signals acquired during the operation control. This poses two challenges: one is the sensors’ addition, which is not attractive due to the increased cost and complexity, and the other is the finding of good features, which usually requires to a great extent the knowledge and experience in signal processing and analysis. In this study, motivated by these issues, a data-driven approach is proposed for the fault diagnosis of electric train doors by using the motor current signals that are used to activate the door operation. A test rig is prepared for the door, in which various fault modes occurring in the train are artificially imbedded, and motor data are collected during the door motion by the communication port. Two approaches are considered for the comparative analyses in the study: one is the traditional features extraction, and the other is the deep learning without resorting to the manual features extraction. In the traditional approach, various time-domain features are extracted using the current signal, from which the optimal features are selected by the combined use of Fisher’s discriminant value (FDV) and Pearson correlation coefficient. Then, the K-nearest neighbor (KNN) algorithm is employed to classify the fault modes. In the deep learning approach, convolutional neural network (CNN) is applied to the motor current signal, which has the advantages of automated features processing and effective implementation by compact architecture [17]. Finally, the classification performances of the two approaches are evaluated by the confusion matrix, in which both show good accuracies in the classification. In this sense, the deep learning approach seems to be superior to the traditional approach, since it does not go through the complex features processing. However, in view of practical applications, it is less useful, as will be explained in Section 5: Practical Application, which addresses how the features are selected and used to monitor the health condition over time during the on-line operation. 

## 2. Data Acquisition

**Figure 1:**
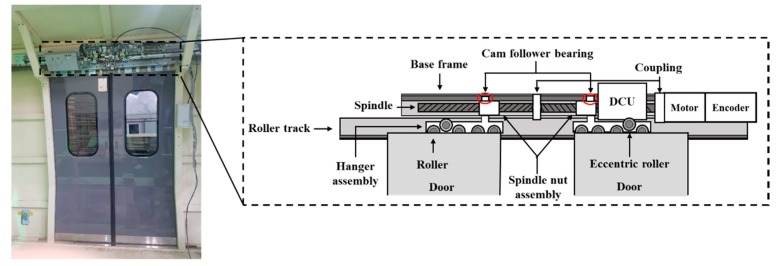
Components of train door test rig.

In this study, an electric door is considered, which consists of the door control unit (DCU), motor, cam follower bearings, rollers, and spindles, as shown in Figure 1. The spindle nut assembly moves linearly along the spindle, in which the cam follower bearing slides within the track of the base frame to prevent the rotation of the assembly. The hanger assembly, which hangs the door, moves along the roller track by the rollers. The hanger assembly and spindle nut assembly are fastened together to move linearly as a single unit along the spindle. Note that there is an eccentric roller in the hanger assembly to prevent vibration during the door movement. The door test rig is provided by the door manufacturing company, which was used for the purpose of durability tests. In order to extract features indicative of fault modes, the current and encoder signal of the motor to control the door operation were used, since they are easily collected through the communication port from the DCU with the sampling rates of 100 Hz and 10 Hz, respectively. 

**Figure 2:**
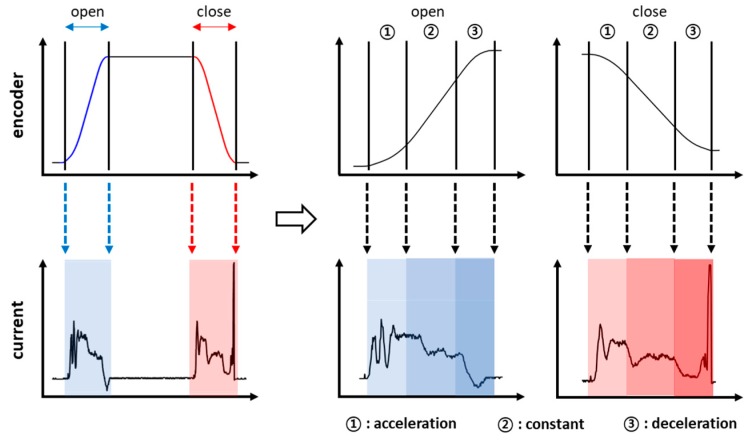
Motor current and encoder signal for the opening and closing operation.

Note that the current signal has been used typically in the fault diagnosis of reciprocating electromechanical mechanisms as found in elevator door systems [8], railway point machines [9], and motor systems [17]. An example of the signal profile is given in Figure 2 during the opening and closing operation, in which the current is supplied to move the door under a given velocity profile, and the rotary angle is measured by the encoder to control the current signal during the motion. As shown in the figure, the current signal can be segmented into three parts in terms of velocity regime: acceleration, constant, and deceleration, each of which can be identified by using the encoder signal.

**Figure 3:**
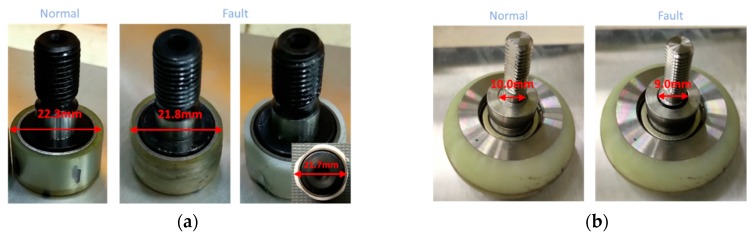
Artificial wear in (**a**) bearing and (**b**) roller.

A total of eight cases are considered for the diagnosis as shown in Table 1, which have been identified as important after consultation with the manufacturer. The extreme condition represents the severe conditions that the door may encounter during the operation. A twisted spindle is imposed such that there is a thin sheet of iron at both ends of each spindle, and they are not parallel with the base frame. An inclined testbed is introduced to replicate the tilting of a train when the train goes through the curved section. A foreign element is imposed by inserting a steel plate between the door and the bottom rail. Natural wear is made to the bearing and roller as a result of a durability test for more than one million cycles of door operation. Artificial wears are also made as shown in Figure 3 to impose greater severity to the cam follower bearing by reducing the diameter by 0.5 mm at the left door and cutting into the triangular shape at the right door, which are shown in the middle and right pictures of Figure 3a respectively, and to the roller by reducing the shaft diameter by 1.0 mm, which creates the gap between the roller and shaft, as shown in Figure 3b. For each case, several data are obtained by repeated operations, with the resulting number of data given in Table 1. The total number of data are 626.

## 3. Feature Extraction-Based Diagnosis

**Figure 4:**
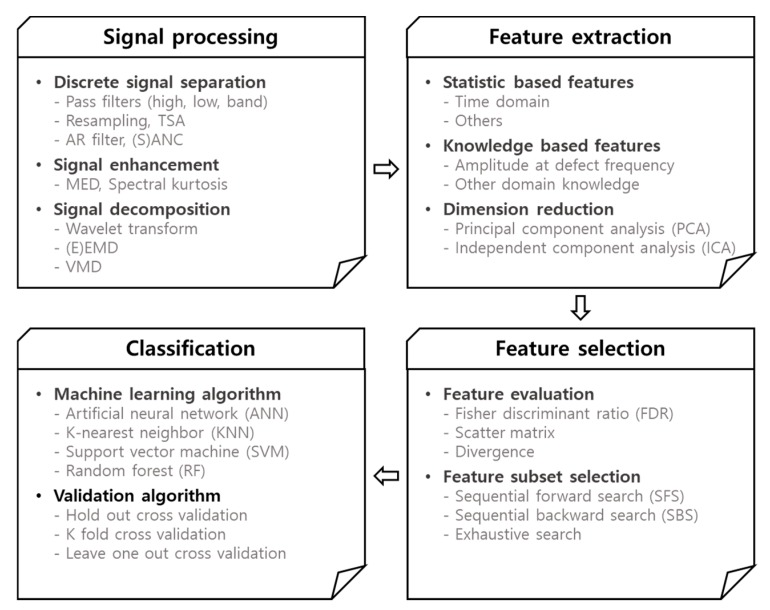
Procedure of traditional features extraction-based diagnosis.

The process of traditional features extraction-based diagnosis is generally composed of four steps as illustrated in Figure 4, which are the signal processing (if necessary), features extraction, features selection, and classification. Since the signal characteristics at the opening and closing operation are different, it is more appropriate to treat them separately in the diagnosis process. Two cases are considered for the signal toward features extraction: one is to use the original signal for the opening and closing operation, respectively. The other is to divide further the signal into three segments by the velocity regimes: acceleration, constant, and deceleration, using the encoder signal, as shown in Figure 2. The features are extracted afterwards for each segmented signal. The boundary of segments is identified by applying the moving average of the time derivative of encoder signal. In the following sections, the diagnosis procedure is illustrated via the original current signal. In the case of the segmented signal, the procedure is the same, differing only by the total number of features increased by three times.

### 3.1. Feature Extraction

In this study, time-domain features, which are one of the most representative ways to identify faults, are extracted from the current signal. A total of 13 features were extracted [18,19], which are given in Table 2.

### 3.2. Feature Selection

Next, a smaller number of features were selected from the extracted features. This is necessary, since too many features increase computations and cause over-fitting. In addition, there may exist duplicate features with high correlation, which should be removed for good separability performance. In order to achieve this, the features are first normalized by their mean and variance. Then, Fisher’s discriminant value (FDV), which is a measure of separability, is calculated [20]:

(1) $$FDV_{l}\left(i,j\right)=\frac{{\left(\mu_{i}-\mu_{j}\right)}^{2}}{\sigma_{i}{}^{2}+\sigma_{j}{}^{2}}$$

where μ and $\sigma^{2}$ denote the mean and variance of the data for class i and j of the feature l. The FDV is calculated for any two classes out of eight, which amounts to 28 combinations. This is obtained for each of 13 features. The results are given in Table 3. From the table, the feature with the highest ranked FDV is chosen for each class combination as listed at the first row with the title ‘Best’. Next, the second feature is chosen with respect to the first one such that the FDV is highest but with the least correlation to avoid the duplicate features. This is sought by the following criteria.

(2) $$k_{2}=arg\underset{m}{\max}\{\alpha {\mathrm{FDV}}_{m}(i,j)-\left(1-\alpha \right)\left|\rho_{k_{1}m}\right|\},\;\;\;\;\;\;\;\;for\;all\;m\neq k_{1}$$

(3) $$\rho_{k_{1}m}=\frac{\sum_{n=1}^{N}x_{k_{1},n}x_{m,n}}{\sqrt{\sum_{n=1}^{N}x_{k_{1},n}{}^{2}\sum_{n=1}^{N}x_{m,n}{}^{2}}}$$

where $k_{1}$ denotes the ID of the first feature, m is the ID other than $k_{1}$, N is the sum number of data of any two classes i and j, and $x_{k_{1},n}$ and $x_{m,n}$ are the individual feature values containing the two classes with IDs $k_{1}$ and m respectively. α is the weight representing the relative importance of the FDV against correlation and set by 0.5 in this study. The $\rho_{k,m}$ is the cross-correlation coefficient for two features with IDs $k_{1}$ and m.

**Figure 5:**
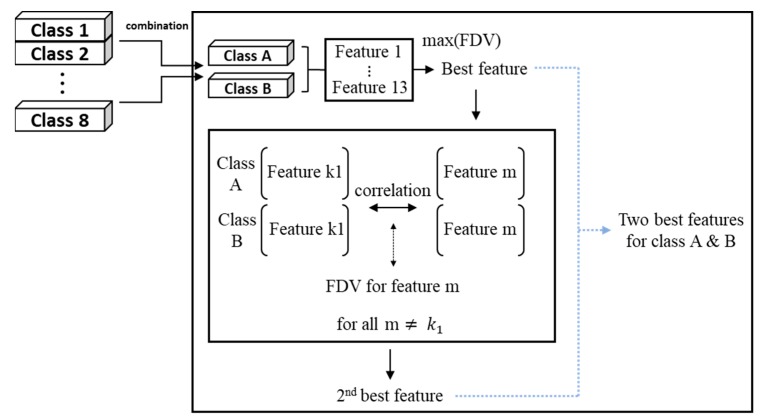
Feature selection procedure by using Fisher’s discriminant value (FDV).

Once the two features are chosen from each combination, these are all gathered to obtain a single group of features, which is given at the bottom row of Table 3 for the opening and closing operation, respectively. While some may overlap with the others among the chosen features, the group represents the collection of features with high FDV and less correlation in terms of two class separability. These are used as the selected features for classification in the next step. The features selection process is summarized in Figure 5.

### 3.3. Classification

**Figure 6:**
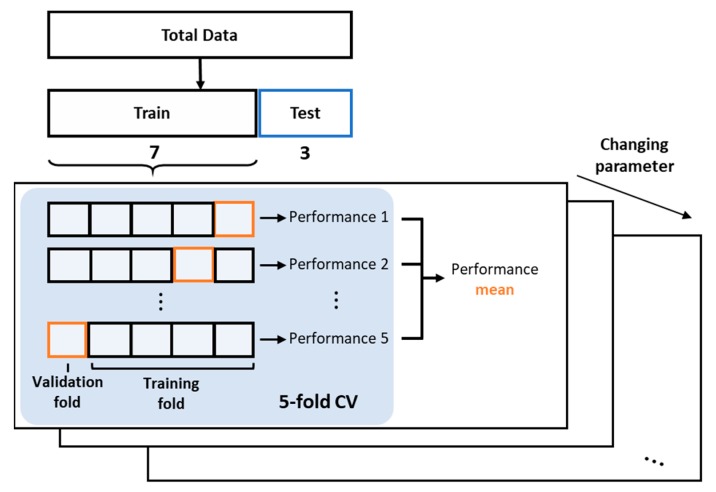
k-fold cross-validation (CV) procedure.

For the classification, the KNN algorithm is applied, which is the simplest and most efficient of the machine learning algorithms. The KNN algorithm calculates the nearest K neighbor of a test data in the training dataset according to the distance measure and assigns the label with the most frequent one. The classification performance depends on the K value such that the model is underfitted for larger K values, whereas it is overfitted for smaller K values. The performance also depends on the way the distance measure is defined, in which the Euclidean and Mahalanobis distance are the most common:

(4) $$d_{\mathrm{Euclidean}(x,y)}=\sqrt{{\left(x-y\right)}^{T}(x-y)}$$

(5) $$d_{\mathrm{Mahalanobis}(x,y)}=\sqrt{{\left(x-y\right)}^{T}\Sigma^{-1}(x-y)}$$

where x and y are the data of selected features from the test and training set, and Σ denotes the covariance matrix made from the training set. Let us define the K value and distance measure as the two hyper-parameters. Then, the k-fold cross-validation (CV) is carried out to explore the proper hyper-parameters. To this end, the data in each condition in Table 1 are divided into the training and test by the ratio of 7:3. As a result, 440 numbers are chosen for the training, whereas the remaining 186 are chosen for the test. The training data are further divided into k equal-sized subsets with k = 5 (note that upper and lowercase letters K and k are used for distinction). Then, the k-fold CV is to train the classifier for the (k − 1) folds using the candidate parameters and evaluate the performance of the classifier to the remaining fold. This is repeated for each fold, and the resulting performances are averaged. The candidate hyper-parameters are the number of neighbors K being from 3 to 11 with the increment of 2, and the distance measure being Euclidean and Mahalanobis. As a result, the best parameters are found to be K = 5 and the Euclidean distance for the both opening and closing operations. The overall k-fold CV process is summarized in Figure 6. Then, the KNN classifier is applied to the test dataset, and the classification performance for each door operation is evaluated using the confusion matrix.

#### 3.3.1. Classification without Current Segmentation

**Figure 7:**
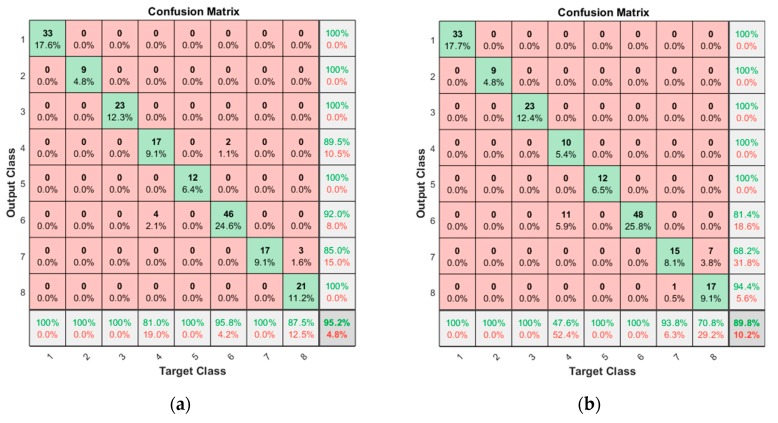
Confusion matrices resulted for non-segmentation: (**a**) result for the opening operation; (**b**) result for the closing operation.

In the case of a current signal without segmentation, the confusion matrices are given in Figure 7, in which the IDs of the row and column represent the output (predicted) and target (true) classes, respectively. The number in each cell is the prediction for the true class. The value beneath is the ratio to the total number of data. The scores at the right end and bottom end are the precision and recall, which represent the ratio of correct classification to the total number of predictions in the row and to the total number of true classes in the column, respectively. The higher precision and recall indicate a better quality of reduced misclassification and misdetection of each fault mode. Finally, the value at the bottom right corner is the accuracy, indicating the ratio of total correct classifications to the total number of data. In this study, this value is adopted for performance evaluation. The results indicate that the accuracy of the open operation is good with 95.2% against the 88.7% for the close operation. The reason for the poor accuracy of the latter is due to the high peak of the current at the end, as shown in Figure 2, which is to ensure that the door is fully closed. This may have caused poor classification performance in the closing operation.

#### 3.3.2. Classification with Current Segmentation

**Figure 8:**
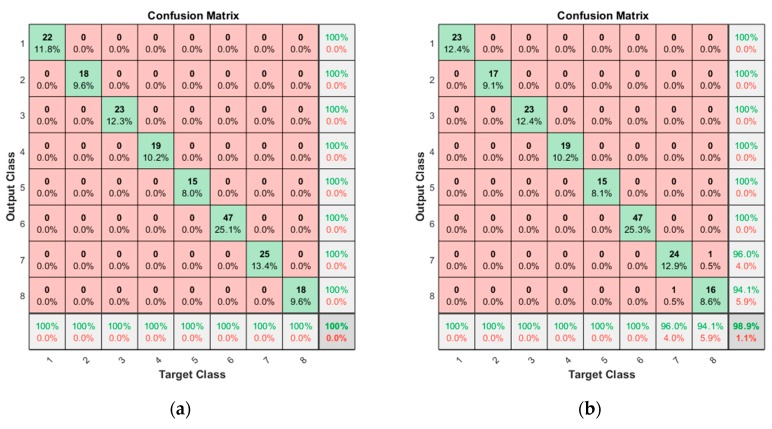
Confusion matrices resulted for segmentation: (**a**) result for the opening operation; (**b**) result for the closing operation.

In the case of current signal with segmentation, in which the extracted features are increased by three times, the confusion matrices are given in Figure 8. The results indicate that the accuracy of the open operation is 100% and 98.9% for the closing operation, which shows remarkable improvement as a result of segmentation. By using the multiple features in the divided segments rather than a single feature in the whole period, much better accuracy is obtained. In the closing operation, two misclassifications are found for the ‘Roller’ (ID 7) and the ’Roller and Bearing’ (ID 8), which means that the roller fault may not be detected easily by this approach. Nevertheless, this is only two out of the whole 186 test data, which is 1%. Except for these, all the classes are perfectly classified.

## 4. Deep Learning-Based Diagnosis

CNN has been widely applied to various fields such as image processing, sound recognition, and fault diagnosis due to the advantage that the classifier is trained with lower parameters than the classical fully connected neural network. The basic architecture of CNN is composed of two main parts: feature extraction and classification. In the feature extraction, features are learned automatically from input raw data. It consists of two layers: convolution and pooling. The convolution layer performs convolutional operation by using a kernel matrix, which should be trained for automatic feature extraction. Then, the pooling layer, also known as the subsampling layer, reduces the dimension of output from the convolution layers by replacing the output of filter with a statistic of the nearby outputs. In the classification, a fully connected MLP and SoftMax layer are executed based on the extracted features, which is the same as the process of an ordinary neural network.

**Figure 9:**
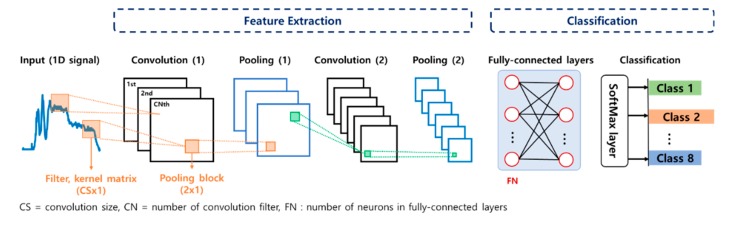
One-dimensional (1D) convolutional neural network (CNN) architecture with a current input.

**Figure 10:**
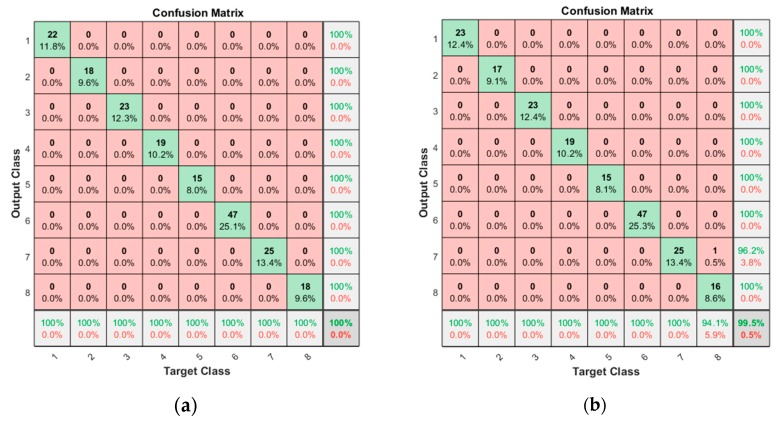
Confusion matrixes resulted from CNN: (**a**) result for the opening operation; (**b**) result for the closing operation.

Since the CNN has been effective in 2D image processing, most of the CNN research studies in the fault diagnosis have dealt with image data such as short time Fourier transform (STFT) or continuous wavelet transform (CWT). However, the CNN is successfully employed for 1D data by replacing a 2D kernel matrix with a 1D matrix. In this study, 1D CNN is applied for the fault diagnosis of train doors using the motor current signals, as shown in Figure 9. More details of the 1D CNN and its architecture can be found in the reference [21]. In the CNN, the Rectified Linear Units (ReLU) function is employed as the activation function, which determines whether the output of each node is activated or not. In addition, max pooling with the 2 × 1 size rectangular block (2 × 1) is used as the pooling function. In order to construct a proper CNN architecture, three parameters are considered for model optimization: convolution size (5, 10, 25, 50), the number of convolution filters (3, 6), and the number of neurons in fully connected layers (50, 100). As in the traditional approach, the k-fold CV is conducted using the training set to find out the parameters with the best performance. As a result, they are found in Table 4. Then, the trained CNN is applied to the test dataset, and the results are given by the confusion matrices in Figure 10. The accuracies from each door operation are 100% for the opening operation and 99.5% for the closing operation. As in the traditional approach, a single misclassification occurs in the closing operation for the class ID 7, which is misinterpreted as 8.

## 5. Practical Application

Comparing the classification accuracies of the CNN and traditional approach, superior accuracy is found in the result by the CNN even with the original current signal without any features processing, which is the greatest advantage in terms of implementation. In the traditional approach, which goes through several steps toward the classification, comparable accuracy is achieved only after the signal is segmented by the velocity regimes. However, the CNN has some drawbacks. Training is time-consuming, and application is limited; it only classifies the faults for the same fault modes and configurations; most importantly, it lacks physical insight. On the other hand, the traditional approach has the advantages in the practical application through the selected features, which are representative of faults. Once established, they can be applied easily to the monitoring of interested faults over time in the real operation.

**Figure 11:**
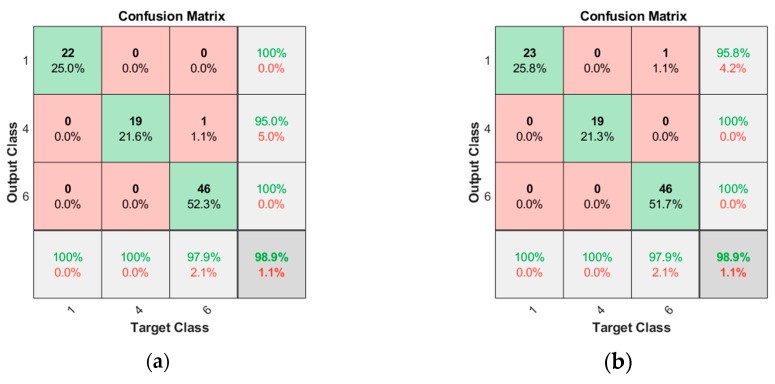
Confusion matrices for three classes by two features: (**a**) 2C and 10C for the open operation; (**b**) 10C and 10D for the close operation.

**Figure 12:**
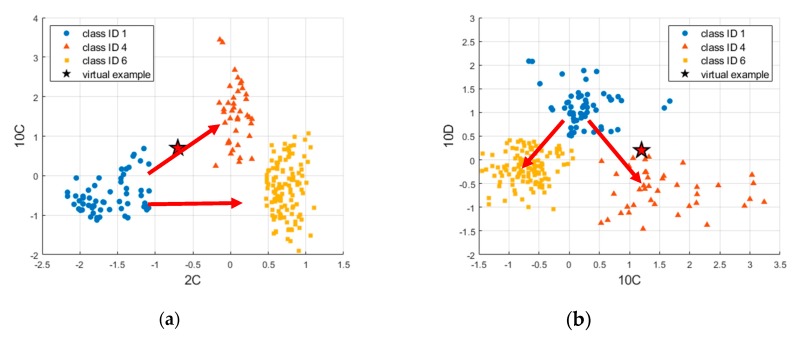
Scatter plots for three classes by two features: (**a**) 2C and 10C for the open operation; (**b**) 10C and 10D for the close operation.

For example, consider classification dealing with the ‘Foreign element insertion’ (ID 4) and ‘Bearing’ (ID 6) faults against the normal state (ID 1), which are the two most frequent fault modes encountered in practice, among those in Table 1. Correct identification of the fault mode is critical, since it prescribes different actions: removing the foreign elements versus replacing the bearing component. Following the same procedure, selected features are given in Table 5, in which the alphabet letters A, C, and D denote the acceleration, constant velocity, and deceleration regime, respectively. After examining the results, the number of features are further reduced to two, which are 2C and 10C, representing the ‘Root mean squares’ and ‘Shape factor’ under constant velocity in the opening operation, and 10C and 10D, representing the ‘Shape factor’ at constant velocity and deceleration in the closing operation, respectively. The confusion matrices by these are given in Figure 11, resulting in a high accuracy of 98.9%. The features data of the normal and the two faults are plotted in Figure 12a,b for the opening and closing operation, respectively. The figures evidently distinguish the two fault states: foreign elements (ID 4) and bearing (ID 6) against the normal (ID 1) by the different directions as are indicated by the arrows in the figures.

The two features can be applied to monitor the development of each fault mode over time in the real operation. Toward this objective, a health index (*HI*) is established by a linear regression model for each fault mode during the opening and closing operations respectively as follows.

(6) $$HI_{ij}\left(x\right)=\alpha_{ij}+\beta_{1,ij}x_{j1}+\beta_{2,ij}x_{j2}$$

where i is the fault mode index of ID 4 and ID 6, j indicates the opening or closing operation, $x_{j1},\;x_{j2}$ denote the two selected features in each operation, and $\alpha_{ij},\;\beta_{1,ij},\;\beta_{2,ij}$ are the coefficients of *HI*s for the *i*th fault at *j*th operation. By taking the features data at the normal and fault conditions and assigning the corresponding *HI*s with 0 and 1 respectively, the coefficients are determined by regression to construct the *HI* model for each fault mode. The results are given in Table 5. Since we have two *HI*s, one can choose the higher *HI*s for conservative purposes or take the average of the two to estimate the current health state by a single value. In this example, the average of the two *HI*s is used. Once the *HI* is obtained, it can be used to monitor the health against each fault mode on-line, which varies between around 0 (normal) and 1 (fault). The overall steps are summarized as follows. 

Off-line development of *HI* from test rig:

1. Acquire current data for normal (ID 1) and seeded faults (ID 4 and 6) in the test rig.
2. Explore two features that can classify the faults for the opening and closing operation, respectively.
3. Establish regression model for *HI* of each fault mode by the two features.

On-line application of *HI* to real operation:

1. Acquire current data during operation.
2. Calculate the *HI* of each fault mode from the current data using the regression model.
3. Take action based on the status between 0 (normal) and 1 (fault) for each fault.

**Figure 13:**
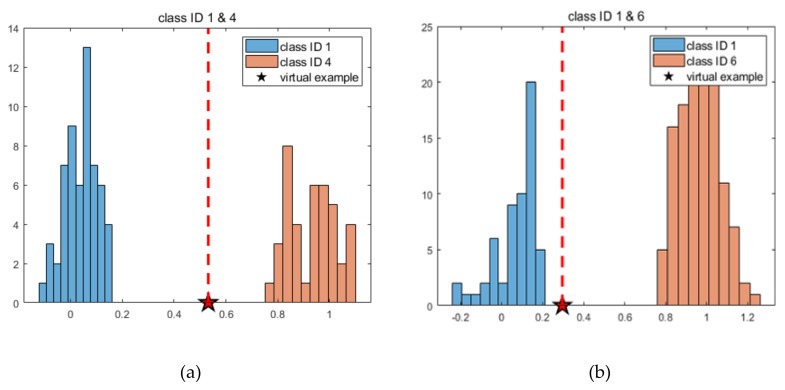
Health index histogram for each fault; (**a**) for class IDs 1 and 4; (**b**) for class IDs 1 and 6.

However, the implementation of this to the real operation is beyond the scope of this paper due to the inability to gather the on-line data. Instead, a virtual example is introduced as an illustration. Assume we have acquired a current signal during the operation, of which the feature values 2C and 10C are −0.7, 0.7 for the opening operation, and 10C and 10D are 1.2, 0.2 for the closing operation. They are given as a star mark in Figure 12. Applying the feature values to the regression model (5), we get *HI* values of 0.5572 and 0.5067 for fault ID 4 and 0.3767 and 0.2152 for fault ID 6, respectively. Take the average of the two *HI*s to estimate the current health state for each fault mode, which are 0.5319 and 0.2959 for ID 4 and 6, respectively. The procedure can also be applied to all the data of the normal of (ID 1) and each seeded fault of (ID 4 and 6) to obtain the *HI* values, which are plotted in the form of a histogram for each fault mode in Figure 13. The current health state is added as star mark in this figure. The value represents that if it is closer to 1, it is highly likely that the corresponding fault will occur, and the train operator or maintainer should make appropriate actions. This is why the traditional approach is advocated over the deep learning approach. 

## 6. Conclusions

In this study, fault diagnosis was carried out with the datasets acquired by operating a train door system of a test rig, in which the datasets included eight different fault modes from the normal and extreme conditions, and the natural and artificial wear of some critical components. In the fault diagnosis, classifications were carried out by dividing the whole data into the training and test data. In the training, k-fold cross-validation was used to find out the optimum architecture of the classifier. Then, the trained classifier was applied to the test dataset, and its accuracy was evaluated, which is the ratio of correct classifications among the whole set. Two approaches were undertaken for comparative purposes: one is the traditional features extraction and the other is the CNN. In the traditional approach, useful features for the classification are sought by applying the FDV and Pearson correlation from the various time statistical features of the current signal. Then, the KNN algorithm is employed to classify the fault modes. Two cases are considered in terms of features: one is to use the original current signal, and the other is to divide further the signal into the three velocity regimes to use the three times larger signals. As a result, in the close operation, the better accuracy of 98.9% is obtained by the signal segmentation as opposed to the 88.7% without it due to the higher discrimination capability at each regime. In the CNN, the best accuracy of 99.5% is found even using the original current signal without any intervention.

The CNN has growing applications in the fault diagnosis; however, it should not be employed without good reason, since it has some drawbacks making them less useful in the real-time implementation: training is time-consuming, it only classifies the faults for the same fault modes and configurations, and it lacks physical insight. On the other hand, the traditional approach has the advantages that a few features can be selected and used to construct the health index (*HI*) of each fault mode to monitor their progressions over time and diagnose during the on-line operations, as was illustrated in Section 5: Practical Application. Therefore, which approach to choose should be determined with discretion according to the desired applications Future study should collect the real current data during the on-line operation over time, use the proposed *HI*s that were applied to assess the health state of the door against each fault, and validate the accuracy by examining the predicted state against the real fault occurrence. 

## Figures and Tables

**Table 1:** Experimental data.

Fault Condition		Number of Data	Class ID
Normal		82	1
Extreme condition	Twisted spindle	46	2
	Twisted spindle and inclined tested	88	3
	Foreign element insertion	66	4
Natural wear	Bearing and roller	46	5
Artificial wear	Bearing	170	6
	Roller	68	7
	Bearing and roller	60	8

**Table 2:** Extracted time domain features.

**Feature ID**	1	2	3	4
**Feature Name**	Mean	Root amplitude	Root meansquares	Standard deviation
**Equation**	$\frac{\sum x_{i}}{N}$	${\left(\frac{\sum \sqrt{\left|x_{i}\right|}}{N}\right)}^{2}$	$\sqrt{\frac{\sum x_{i}{}^{2}}{N}}$	$\sqrt{\frac{\sum {\left(x_{i}-\bar{x}\right)}^{2}}{N-1}}$
**Feature ID**	5	6	7	8
**Feature Name**	Peak	Skewness	Kurtosis	Crest factor
**Equation**	max(x)	$\frac{\sum {\left(x_{i}-\bar{x}\right)}^{3}}{(N-1){\left(\sqrt{\frac{\sum {\left(x_{i}-\bar{x}\right)}^{2}}{N-1}}\right)}^{3}}$	$\frac{\frac{1}{N}\sum {\left(x_{i}-\bar{x}\right)}^{4}}{{\left(\frac{1}{N}\sum {\left(x_{i}-\bar{x}\right)}^{2}\right)}^{2}}$	$\frac{\left|x_{peak}\right|}{x_{rms}}$
**Feature ID**	9	10	11	12
**Feature Name**	Clearance factor	Shape factor	Impulse factor	Peak-to-peak
**Equation**	$\frac{x_{peak}}{{\left(\frac{\sum \sqrt{\left|x_{i}\right|}}{N}\right)}^{2}}$	$\frac{x_{rms}}{\frac{1}{N}\sum \left|x_{i}\right|}$	$\frac{x_{peak}}{\frac{1}{N}\sum \left|x_{i}\right|}$	max(x)−min(x)
**Feature ID**	13			
**Feature Name**	Root sum of squares			
**Equation**	$\sqrt{\sum x_{i}{}^{2}}$			

**Table 3:** Selected features for each operation.

**Open Operation**								
Class Order (Combination)		Class 1&2	Class 1&3	Class 1&4	Class 1&5	Class 1&6	Class 1&7	Class 1&8
Feature ID	Best	3	3	1	2	3	3	1
	2nd Best	13	13	13	1	13	13	13
Class Order Combination)		Class 2&3	Class 2&4	Class 2&5	Class 2&6	Class 2&7	Class 2&8	Class 3&4
Feature ID	Best	3	4	10	3	10	10	1
	2nd Best	13	3	4	13	2	2	2
Class Order (Combination)		Class 3&5	Class 3&6	Class 3&7	Class 3&8	Class 4&5	Class 4&6	Class 4&7
Feature ID	Best	2	2	3	1	2	1	13
	2nd Best	1	1	13	13	10	2	3
Class Order (Combination)		Class 4&8	Class 5&6	Class 5&7	Class 5&8	Class 6&7	Class 6&8	Class 7&8
Feature ID	Best	1	2	13	13	3	1	3
	2nd Best	13	1	3	3	13	13	13
Gathered Features		1, 2, 3, 4, 10, 13						
**Close Operation**								
Class Order (Combination)		Class 1&2	Class 1&3	Class 1&4	Class 1&5	Class 1&6	Class 1&7	Class 1&8
Feature ID	Best	6	10	2	2	2	2	2
	2nd Best	9	9	1	1	1	1	1
Class Order (Combination)		Class 2&3	Class 2&4	Class 2&5	Class 2&6	Class 2&7	Class 2&8	Class 3&4
Feature ID	Best	6	6	11	6	6	6	1
	2nd Best	1	9	6	11	11	11	2
Class Order (Combination)		Class 3&5	Class 3&6	Class 3&7	Class 3&8	Class 4&5	Class 4&6	Class 4&7
Feature ID	Best	1	10	1	1	2	2	2
	2nd Best	2	4	2	2	1	1	1
Class Order (Combination)		Class 4&8	Class 5&6	Class 5&7	Class 5&8	Class 6&7	Class 6&8	Class 7&8
Feature ID	Best	2	2	2	2	1	1	6
	2nd Best	1	1	1	1	2	2	7
Gathered Features		1, 2, 4, 6, 7, 9, 10, 11						

**Table 4:** Optimum parameters of CNN architecture.

	Open	Close
Convolution size	25	50
Number of convolution filter	6	6
Number of neurons in fully connected layers	100	100

**Table 5:** Selected features for class 1, 4, and 6 at each door operation.

Open Operation					Close Operation				
Class Order		Class	Class	Class	Class Order		Class	Class	Class
(Combination)		1&4	1&6	4&6	(Combination)		1&4	1&6	4&6
Feature ID	Best	3C	2C	2C	Feature ID	Best	10C	3C	10C
	2nd Best	10C	5D	12C		2nd Best	10D	1C	6D
Gathered Features		2C, 3C, 10C, 12C, 5D			Gathered Features		1C, 3C, 10C, 6D, 10D		
